# Sulfur-Functionalized *N*-Heterocyclic Carbene Complexes of Pd(II): Syntheses, Structures and Catalytic Activities

**DOI:** 10.3390/molecules17032491

**Published:** 2012-03-01

**Authors:** Dan Yuan, Han Vinh Huynh

**Affiliations:** Department of Chemistry, 3 Science Drive 3, National University of Singapore, 117543, Singapore; Email: g0700466@nus.edu.sg

**Keywords:** N-heterocyclic carbenes, sulfur functions, Pd(II) complexes, catalysis

## Abstract

N-heterocyclic carbenes (NHCs) can be easily modified by introducing functional groups at the nitrogen atoms, which leads to versatile coordination chemistry as well as diverse catalytic applications of the resulting complexes. This article summarizes our contributions to the field of NHCs bearing different types of sulfur functions, *i.e.*, thioether, sulfoxide, thiophene, and thiolato. The experimental evidence for the truly hemilabile coordination behavior of a Pd(II) thioether-NHC complex has been reported as well. In addition, complexes bearing rigid CSC-pincer ligands have been synthesized and the reasons for pincer *versus* pseudo-pincer formation investigated. Incorporation of the electron-rich thiolato function resulted in the isolation of structurally diverse complexes. The catalytic activities of selected complexes have been tested in Suzuki-Miyaura, Mizoroki-Heck and hydroamination reactions.

## 1. Introduction

N-heterocyclic carbenes (NHCs) are nowadays ubiquitous ligands in organometallic chemistry as well as catalysis due to their unique properties [1]. Being strong σ-donors and generally weak π-acceptors, NHCs in general form strong bonds with varieties of metal centers, which results in highly stable complexes. Moreover, both steric and electronic properties of NHCs can be conveniently tuned by changing the substituents and backbones, which also contributes to their popularity. Modification of NHCs can be easily achieved by introducing functional groups at the nitrogen atoms [2,3,4]. Donor-functionalized NHCs are potentially polydentate ligands, which can give rise to complexes with enhanced stability through ligand chelation. Varieties of functionalized NHC complexes bearing bidentate chelating NHCs, tripodal NHCs and pincer-type NHCs have been reported and tested in catalysis [2,3,4]. Since carbenes form strong M-C bonds (*vide supra*), the presence of other donors that form weaker bonds with metal centers makes these polydentate ligands potentially hemilabile. The concept of hemilability has become important in catalyst design as hemilabile ligands have the ability to provide free coordination sites for substrate activation at the metal center during catalysis, or to stabilize catalytically active species [5].

**Figure 1:**
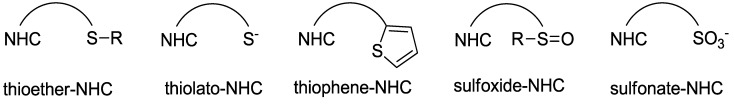
NHCs with different sulfur functions.

While NHCs with N, O or P donors are relatively common [2,3,4], those with sulfur donors still remain relatively unexplored. The sulfur atom exhibits different oxidation states ranging from −2 to +6, which enriches the already versatile NHC chemistry. NHCs with sulfur functions can be mainly categorized into thioether-NHC, thiolato-NHC, sulfonate-NHC, thiophene-NHC, and sulfoxide-NHC (Figure 1) [6]. In this review, we will describe our contribution to the preparation of NHC Pd(II) complexes with different sulfur functions, their structures and catalytic applications.

## 2. Sulfur-Functionalized Azolium Salts

Azolium salts bearing a range of sulfur-functions, *i.e.*, thioether [7,8,9,10], sulfoxide [9], thioester [11,12], thiol [11], and thiophene [13], have been synthesized as precursors to S-functionalized NHC complexes. 

### 2.1. Thioether-Functionalized Azolium Salts

**Scheme 1:**
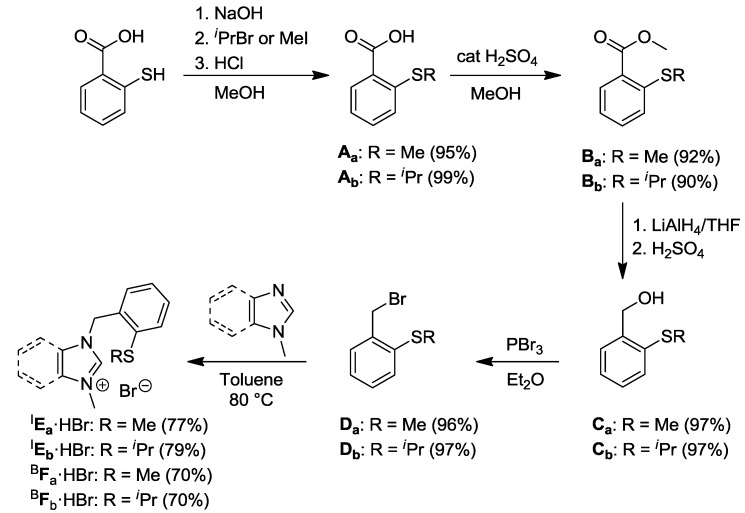
Syntheses of alkyl-aryl thioether-functionalized azolium salts [7,8].

The alkyl-aryl thioether functionalized imidazolium (^I^**E_a/b_·**HBr) and benzimidazolium (^B^**F_a/b_·**HBr) salts were synthesized in a five-step sequence (Scheme 1) [7,8]. Commercially available thiosalicyclic acid was treated with alkyl bromides to form the 2-alkylmercaptobenzoic acids **A_a/b_**. Subsequent esterification of **A_a/b_** in MeOH in the presence of H_2_SO_4_ yielded the mercaptobenzoates **B_a/b_**, which were reduced with LiAlH_4_ under anhydrous conditions and acidified to afford the alcohols **C_a/b_**. The benzyl bromides **D_a/b_** were eventually obtained from the substitution reaction of **C_a/b_** with PBr_3_. N-alkylation of *N*-methylimidazole and *N*-methylbenzimidazole gave rise to the respective thioether functionalized carbene precursors in overall yields of 63% (^I^**E_a_·**HBr), 64% (^I^**E_b_·**HBr), 57% (^B^**F_a_·**HBr), and 59% (^B^**F_b_·**HBr) over five steps.

### 2.2. Thioether-Bridged Diazolium Salts

**Scheme 2:**
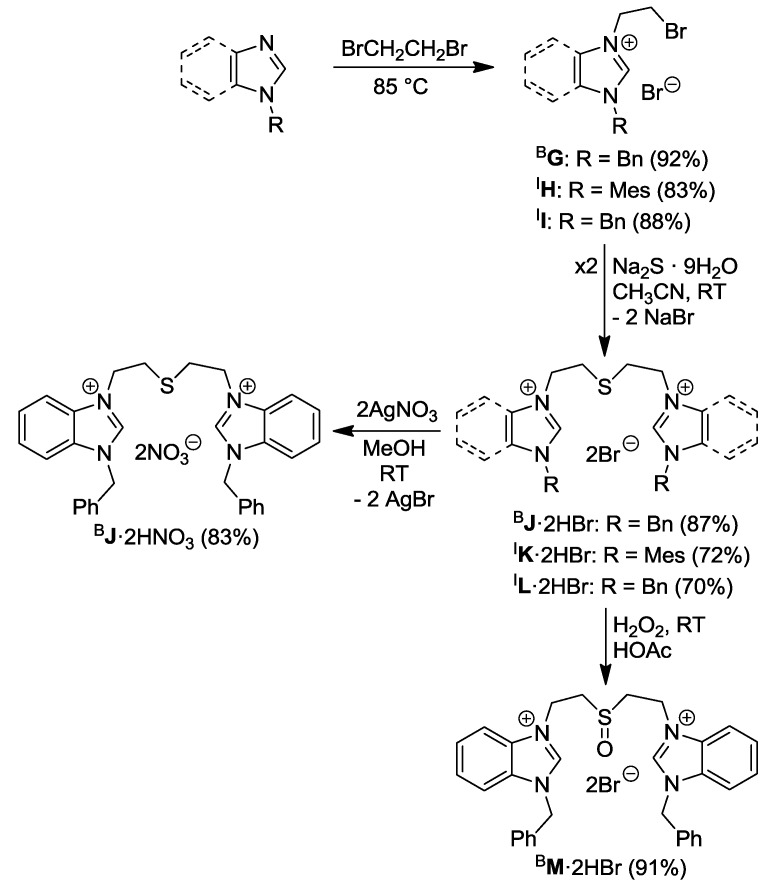
Syntheses of thioether/sulfoxide-bridged azolium salts [9,10].

The alkyl-alkyl thioether-bridged diazolium salts, on the other hand, were synthesized in a different approach (Scheme 2) [9,10]. 

The reaction of benzylbenzimidazole with neat dibromoethane afforded 1-benzyl-3-bromoethylbenzimidazolium bromide ^B^**G**, which is a useful precursor to salts with different functional groups (*vide infra*). Two equiv of ^B^**G** underwent nucleophilic substitution with Na_2_S to form the thioether-bridged dibenzimidazolium dibromide ^B^**J**·2HBr. Anion exchange occurred smoothly by reacting ^B^**J**·2HBr with two equiv of AgNO_3 _affording ^B^**J**·2HNO_3_ with the precipitation of AgBr. In addition, the sulfoxide-bridged dibenzimidazolium salt ^B^**M**·2HBr was prepared by oxidation of the thioether ^B^**J**·2HBr with three equiv of H_2_O_2_ in acetic acid at ambient temperature.

Similarly, the thioether-bridged diimidazolium salts ^I^**K**·2HBr and ^I^**L**·2HBr can be synthesized from N-substituted imidazoles in two steps (Scheme 2). The first step involved quaternation of 1-mesitylimidazole and 1-benzylimidazole with neat 1,2-dibromoethane affording salts ^I^**H** and ^I^**I**, which were treated with Na_2_S giving rise to thioether-bridged ligand precursors ^I^**K**·2HBr and ^I^**L**·2HBr. 

**Scheme 3:**
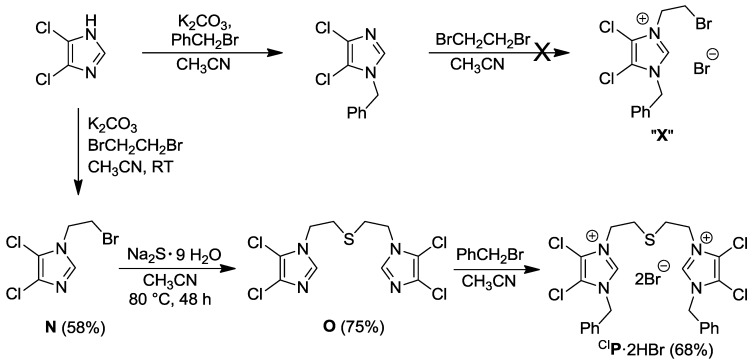
Synthesis of thioether-bridged 4,5-dichlorodiimidazolium salt ^CI^**P**·2HBr [10].

This successful strategy for the synthesis of ^I^**K**·2HBr, however, failed in the preparation of the electron poorer 4,5-dichlorodiimidazolium-analogue ^CI^**P**·2HBr (Scheme 3). Apparently, 1-benzyl-4,5-dichloroimidazole is too electron deficient to be alkylated by 1,2-dibromoethane to afford the illusive species “**X**”.

To circumvent this problem, another synthetic route was developed (Scheme 3) [10], in which the thioether-bridge was installed first followed by quaternation with the stronger electrophile benzylbromide. Thus, 4,5-dichloroimidazole was first alkylated with 1,2-dibromoethane to yield 1-bromoethyl-4,5-dichloroimidazole **N**, which was then treated with Na_2_S to form the sulfur-bridged diimidazole **O**. Alkylation of **O** with benzylbromide finally afforded the target compound ^CI^**P**·2HBr. 

### 2.3. Thioester- and Thiol-Functionalized Azolium Salts

**Scheme 4:**
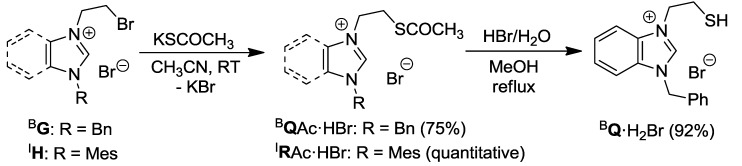
Syntheses of thioester/thiol-functionalized azolium salts [11,12].

Nucleophilic substitution of salt ^B^**G** with 1.2 equiv of KSCOCH_3_ in CH_3_CN afforded the thioester-functionalized benzimidazolium salt ^B^**Q**Ac·HBr (Scheme 4) [11]. In a similar manner, imidazolium analogue ^I^**R**Ac·HBr was obtained as a brown oil from ^I^**H** [12]. The thioester group of ^B^**Q**Ac·HBr was easily hydrolyzed by aqueous HBr to form the thiol-functionalized salt ^B^**Q**·H_2_Br. 

### 2.4. Thiophene-Functionalized Azolium Salt

**Scheme 5:**
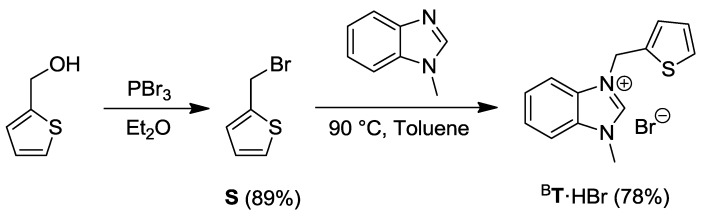
Synthesis of the thiophene-functionalized benzimidazolium salt ^B^**T**·HBr [13].

The thiophene-functionalized benzimidazolium bromide ^B^**T**·HBr was synthesized from the commercially available 2-thiophenemethanol, which was brominated with PBr_3_ and heated with *N*-methylbenzimidazole to afford the desired compound (Scheme 5) [13].

## 3. Thioether-NHC Complexes

The thioether group is the most common among the sulfur-functions described in this work [6,14]. Using thioether-functionalized NHCs and diNHCs a range of bis(carbene), monocarbene, CSC pincer and pseudopincer complexes of palladium have been obtained, which are described in the following paragraphs in more detail. Notably, the first experimental evidence for the true hemilabile coordination behavior of a donor-functionalized NHC was presented using a thioether-NHC Pd(II) complex.

### 3.1. Thioether-Functionalized Pd(II) Bis(carbene) Complexes

**Scheme 6:**
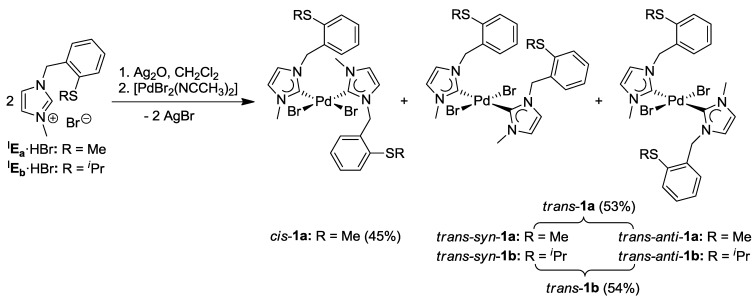
Syntheses of thioether-functionalized Pd(II) bis(imidazolin-2-ylidene) complexes [7,8].

The complexation of two equiv of ^I^**E_a_·**HBr with Pd(OAc)_2_ in acetonitrile at 90 °C gave rise to a complex product mixture, from which only *cis*-[PdBr_2_(^I^**E_a_**)_2_] (*cis*-**1a)** could be isolated in a low yield of 38% [7,8]. The difficulty of carbene formation and subsequent coordination to the Pd(II) center can be attributed to competing processes such as (i) nonselective coordination of the soft sulfur atom, (ii) competing deprotonation of C4/C5- or benzylic protons. Thus the alternative and milder Ag-carbene transfer method was explored (Scheme 6) [15]. Two equiv of ^I^**E_a_·**HBr were treated with Ag_2_O in CH_2_Cl_2_, and the resulting Ag-carbene species was added into an CH_3_CN solution of [PdBr_2_(CH_3_CN)_2_]. As expected, an isomeric mixture of bis(carbene) complexes *cis*-**1a **(45%), *trans*-*anti*-**1a **(27%), and *trans*-*syn*-**1a** (27%) was obtained in an improved yield of 98%.

Similar reaction conditions were applied for the complexation of imidazolium salt ^I^**E_b_·**HBr, and an approximately 1:1 mixture of *trans-anti*-[PdBr_2_(^I^**E_b_**)_2_] (*trans*-*anti*-**1b)** and *trans*-*syn*-**1b** was isolated (Scheme 6). Surprisingly, *cis*-isomers were not detected. 

**Scheme 7:**
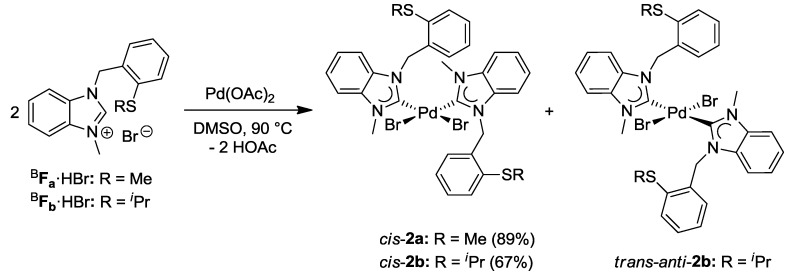
Syntheses of thioether-functionalized Pd(II) bis(benzimidazolin-2-ylidene) complexes [8].

Due to the absence of additional acidic protons on the heterocyclic backbone, the benzimidazolium salts ^B^**F_a_·**HBr and ^B^**F_b_·**HBr reacted more cleanly with Pd(OAc)_2_ compared to their imidazolium analogues, giving rise to *cis*-configured complexes *cis*-[PdBr_2_(^B^**F_a/b_**)_2_] (*cis*-**2a**/**b**) in yields of 89% and 67%, respectively (Scheme 7) [8].

**Figure 2:**
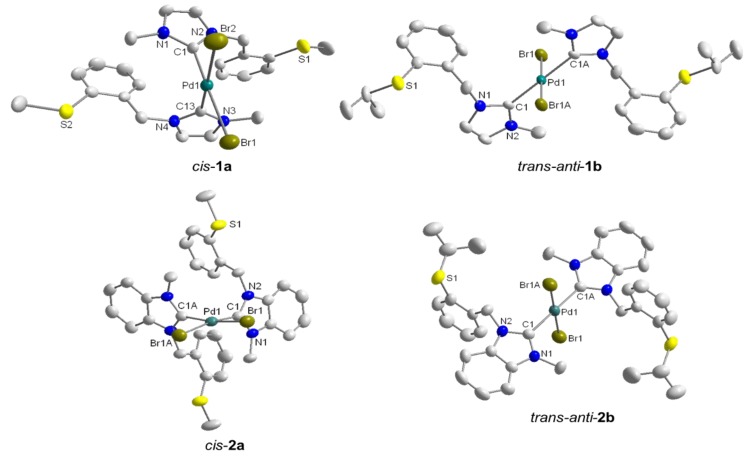
Molecular structures of *cis*-**1a**, *trans*-*anti*-**1b**, *cis*-**2a** and *trans*-*anti*-**2b** [8].

The molecular structures of *cis*-**1a**, *trans*-*anti*-**1b**, and *cis*-**2a** have been obtained from X-ray diffraction analysis on single crystals (Figure 2). In addition, *cis*-*trans* isomerization of *cis*-**2b** occurred upon crystallization, which gave rise to the solid state structure of *trans*-*anti*-**2b**. All structures show that each Pd(II) center adopts a square-planar geometry, coordinated by two NHCs and two bromido ligands, while the thioether functions remain pendant. Overall, it was observed that *cis*-isomers preferably crystallized when R = CH_3_, while crystals of *trans*-isomers form when R = CH(CH_3_)_2_. It is interesting to note that changes remote from the metal center can trigger *cis-trans* isomerization in these bis(carbene) complexes.

### 3.2. Hemilabile Thioether-Functionalized Pd(II) Monocarbene Complexes

**Figure 3:**
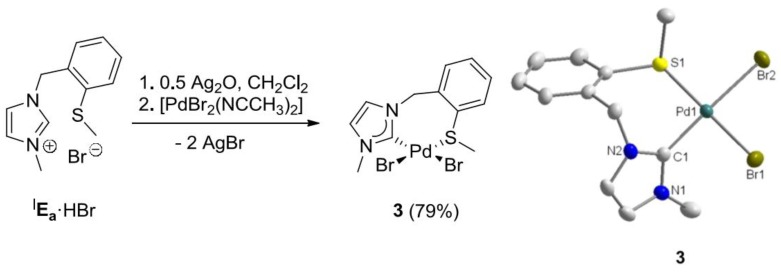
Synthesis of Pd(II) monocarbene complex **3** and its molecular structure [7,8].

**Figure 4:**
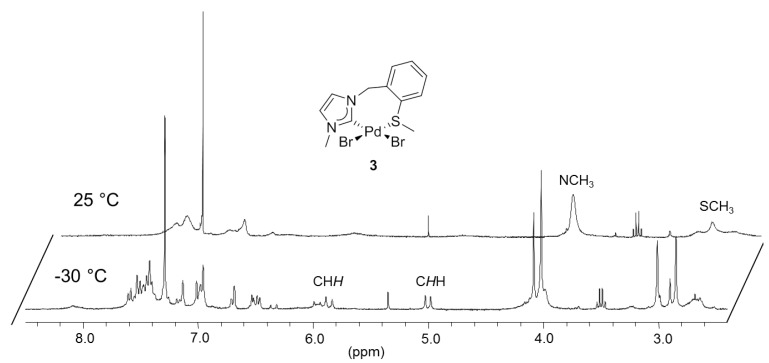
^1^H -NMR spectrum of **3** (300 MHz, CDCl_3_).

To synthesize monocarbene complexes, which should allow thioether coordination, the Ag-carbene transfer reaction with a 1:1 stoichiometric ratio of ^I^**E_a_·**HBr to Pd was employed (Figure 3) [7,8]. The ^1^H-NMR spectrum of the reaction product in CDCl_3_ shows very broad signals at ambient temperature (Figure 4). Upon cooling to −30 °C, two doublets at 5.00 and 5.85 ppm were resolved for the nonequivalent benzylic protons, which indirectly indicate coordination of the sulfur function. The identity of complex *cis*-[PdBr_2_(^I^**E_a_-**k^2^*C,S*)] (**3**) was finally confirmed by X-ray diffraction, which shows that the carbene and the sulfur atom coordinate to the metal center in a *cis*-chelating manner, resulting in a seven-membered ring (Figure 3). The newly formed Pd-S bond amounts to 2.3079(3) Å.

**Scheme 8:**
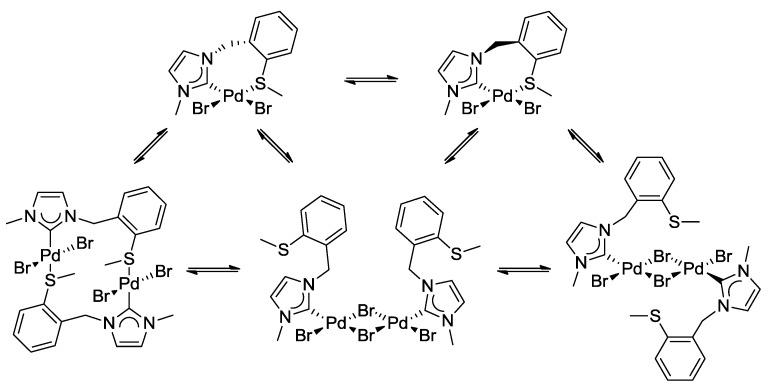
Dynamic process due to the hemilabile ligand in complex **3**.

The complicated ^1^H-NMR spectrum of complex **3** was mainly attributed to two dynamic processes that occur in solution (Scheme 8). The first process involves flipping of the seven-membered ring, and syn- and anti-isomerization, which slows down upon cooling, giving rise to the inequivalent benzylic protons observed at low temperature.

The second process involves reversible de- and recoordination of the sulfur donor in a hemilabile fashion. Decoordination results in a formally unsaturated metal center, which stabilizes itself upon dimerization. The two dynamic processes are in equilibrium with potentially at least five different species. The situation is further complicated due to the fact that the thioether function becomes a chiral center upon coordination.

**Figure 5:**
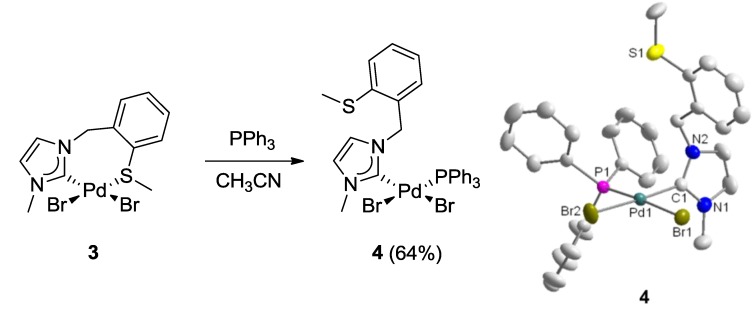
Synthesis of Pd(II) monocarbene complex **4** and its molecular structure [7,8].

**Figure 6:**
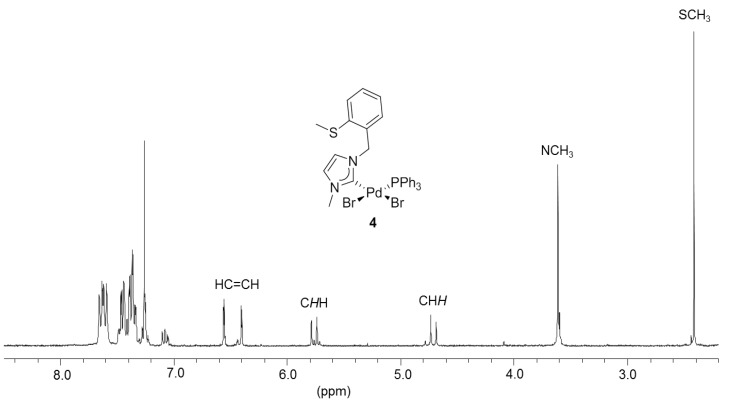
^1^H-NMR spectrum of **4 **(300 MHz, CDCl_3_).

The hemilabile behavior of the thioether-NHC ligand was finally confirmed by the addition of one equiv (based on Pd) of PPh_3_ to the solution of **3** (Figure 5) [7,8]. The stronger phosphine donor is expected to cleave potential dimeric complexes as well as the weaker Pd-S bond in **3**. Indeed, the addition of PPh_3_ led to the formation of the mixed NHC-phosphine complex *cis*-[PdBr_2_(^I^**E_a_**)(PPh_3_)] (**4**) as the sole product, as evidenced by clean and well-resolved multi-nuclear NMR spectra (Figure 6) as well as X-ray diffraction analysis (Figure 5).

## 4. CSC-Pincer Type Pd(II) Complexes

Most known pincer systems contain rather rigid structures, which are intended to yield increased complex stability [16,17]. On the other hand, a more flexible ligand backbone would allow a more subtle interplay between lability and stability, which may be beneficial for certain types of catalytic applications. Thus, we investigated the syntheses of Pd(II) complexes with CSC-pincer type ligands using thioether-bridged diazolium salts.

### 4.1. Pd(II) Benzimidazolin-2-ylidene Complexes

**Scheme 9:**
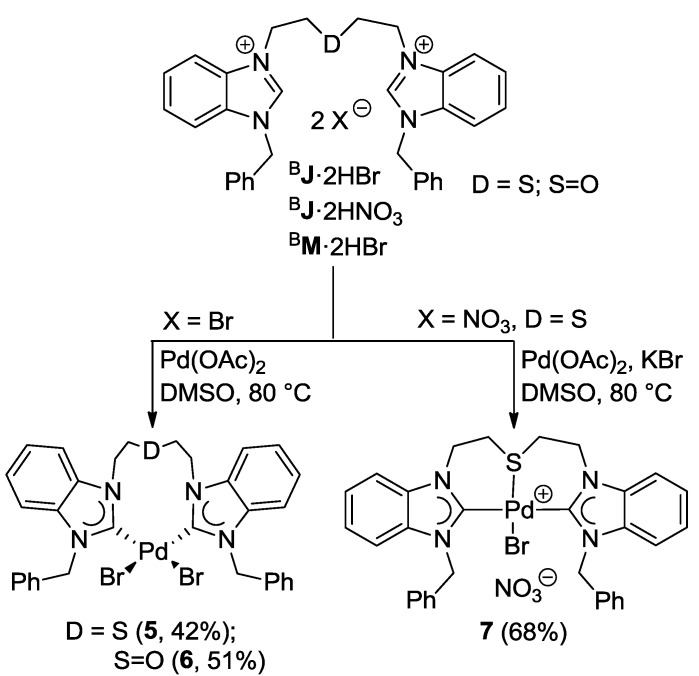
Syntheses of Pd(II) complexes with benzimidazolin-2-ylidene CSC-pincer type ligands [9].

**Figure 7:**
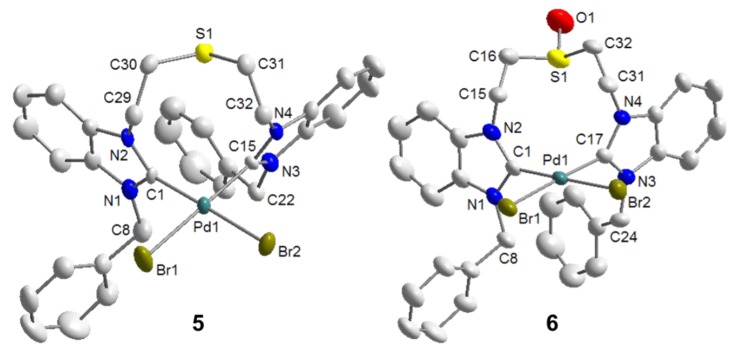
Molecular structures of **5** and **6** [9].

Palladation of the thioether-bridged benzimidazolium salt ^B^**J**·2HBr with Pd(OAc)_2_ led to the pseudo-pincer dicarbene complex *cis*-[PdBr_2_(^B^**J-***k*^2^*C*)] (**5**), in which the thioether function remains pendant (Scheme 9) [9]. Similarly, the reaction of precursor ^B^**M**·2HBr gave an analogous complex *cis*-[PdBr_2_(^B^**M-***k*^2^*C*)] (**6**), which is to date the only example of a complex bearing a sulfoxide-functionalized NHC ligand. The pseudo-pincer configurations of both complexes have been confirmed by their solid state structures (Figure 7).

In an attempt to overcome the formation of pseudo-pincer complexes and to enforce a CSC-pincer-type coordination mode of ligand ^B^**J**, ligand precursor ^B^**J**·2HBr was substituted with ^B^**J**·2HNO_3_. The latter only contains weakly coordinating counter-anions, which should facilitate binding of the soft thioether donor. Disappointingly, the equimolar reaction of ^B^**J**·2HNO_3_ with Pd(OAc)_2_ in DMSO at 80 °C only led to substantial formation of Pd black.

**Figure 8:**
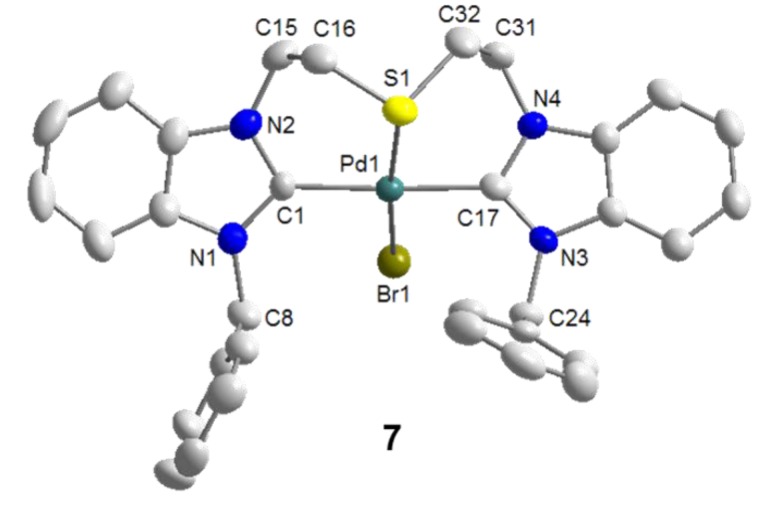
Molecular structure of **7**. The non-coordinating counter-anion NO_3_^-^ has been omitted [9].

It was anticipated that the lack of stabilizing anionic ligands was the cause for this decomposition. Indeed with the addition of 1 equiv of KBr, which provides a bromido ligand to take up the fourth coordination site, the reaction proceeded smoothly and the desired product *trans*-[PdBr(^B^**J-***k*^3^*CSC*)]NO_3_ (**7**) could be isolated in a good yield of 68% (Scheme 9). The solid state structure of **7** shows that the pincer-type coordination forces the two NHCs *trans* to each other, and the bromido ligand indeed binds to the Pd(II) center *trans* to the thioether donor (Figure 8). The Pd-S bond of 2.3078(18) Å is comparable to that of monocarbene complex **3 **(*vide supra*). 

### 4.2. Pd(II) Imidazolin-2-ylidene Complexes

Although imidazolin-2-ylidenes are the most studied types of NHCs, their Pd(II) CSC-pincer complexes were unknown. Since the methodologies of synthesizing benzimidazole-based ligand precursors and complexes are straightforward (*vide supra*), an extension to imidazolin-2-ylidene systems was carried out [10].

**Scheme 10:**
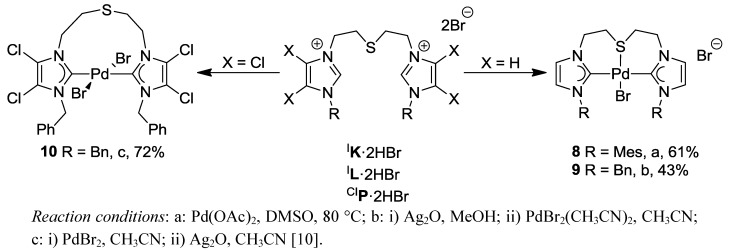
Syntheses of CSC pincer complexes **8**, **9** and pseudo-pincer complex **10**.

Palladation of thioether-bridged imidazolium salt ^I^**K·**2HBr with Pd(OAc)_2_ afforded the cationic pincer complex *trans*-[PdBr(^I^**K-***k*^3^*CSC*)]Br (**8**) with a bromide counter-anion (Scheme 10). Its formation can be attributed to the bulky mesityl substituents that enforce a *trans* configuration of the carbene donors, which in turn facilitates the coordination of the sulfur atom to the Pd(II) center enhancing the chelate effect of the CSC pincer ligand.

**Figure 9:**
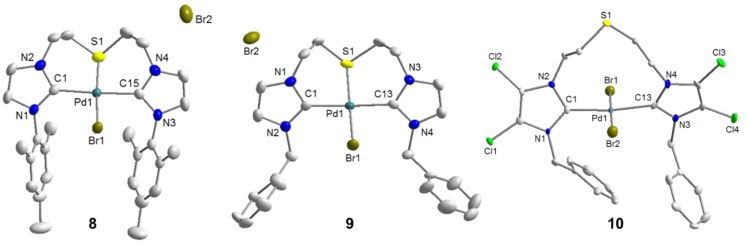
Molecular structures of **8**, **9** and **10** [10].

In order to study the steric influence in more detail, diimidazolium salt ^I^**L·**2HBr carrying less bulky and more flexible benzyl groups was subjected to complexation. Using the Ag-carbene transfer route, ^I^**L·**2HBr was converted to the pincer complex *trans*-[PdBr(^I^**L**-*κ*^3^*CSC*)]Br (**9**) (Scheme 10), and its solid state molecular structure determined by X-ray diffraction analysis on single crystals is shown in Figure 9. 

The straightforward formation of CSC pincer **9** from diimidazolium dibromide ^I^**L·**2HBr requires further comment, since palladation of the direct dibenzimidazolium analogue resulted in a neutral *cis*-dibromido-dicarbene complex **5** with dangling thioether function (*vide supra*). Since imidazolin-2-ylidenes are generally stronger donors than benzimidazole analogues [18], it appears that the donor strength of carbenes influences pincer *versus* pseudo-pincer formation. Stronger donating carbenes may favor pincer formation even in the presence of halide ions, whereas weaker carbenes prefer to form neutral pseudo-pincer complexes (*vide infra*). In the latter case, pincer formation can be enforced by using salt precursors with non- or only weakly coordinating anions.

To find further proof for this concept, the synthesis of a Pd(II) complex bearing weakly donating 4,5-dichloroimidazolin-2-ylidenes derived from ^CI^**P·**2HBr was carried out (Scheme 10). The ligand precursor ^CI^**P·**2HBr was first treated with PdBr_2_ to yield a palladate, which was converted to a carbene complex with the addition of Ag_2_O. Based on our hypothesis, a pseudo-pincer complex was anticipated. Indeed the pseudo-pincer complex *trans*-[PdBr_2_(^CI^**P-***k*^2^*C*)] (**10**) was isolated, which was fully characterized by multinuclei NMR spectroscopies and X-ray analysis. The solid state structure depicts a *trans*-spanning dicarbene ligand with a pendant thioether function (Figure 9).

In the present case, we propose that the polarization between carbenes and the Pd(II) center is enhanced with stronger carbene donors (e.g., in **8** and **9**), which leads to a pile-up of electron density at the bromido ligands making them a better leaving group, and eventually rendering the Pd(II) center more Lewis acidic for the coordination of the thioether donor. With weaker NHC donors (e.g., in **5**, **6** and **10**) such strong polarization is less likely, which results in a neutral pseudo-pincer complexes. In other words, stronger donors stabilize cationic pincer complexes more efficiently than weaker donors.

## 5. Thiophene-NHC Complexes

**Figure 10:**
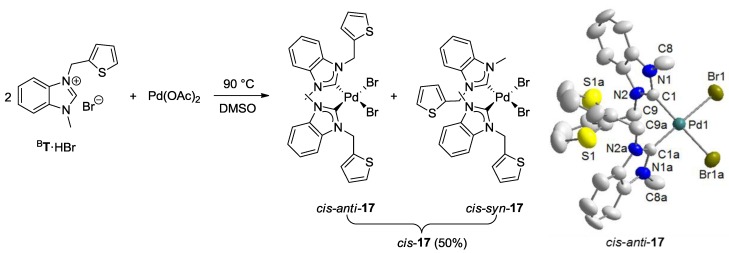
Synthesis of thiophene-functionalized Pd(II) complex *cis*-**17** and its molecular structure [13].

**Figure 11:**
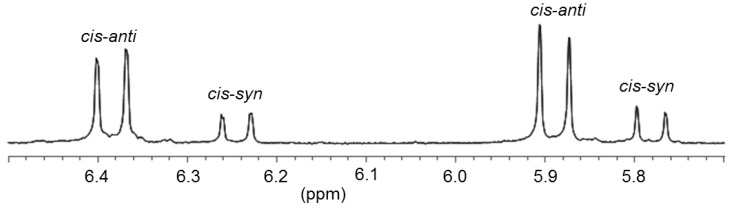
Diastereotopic ^1^H-NMR signals of NCH_2_ protons in *cis-anti* and *cis-syn* rotamers of *cis*-**17** (500 MHz, CD_2_Cl_2_) [13].

Reaction of two equiv of thiophene-functionalized salt ^B^**T**·HBr with Pd(OAc)_2 _afforded the desired bis(carbene) complex *cis*-**17** (Figure 10) [13]. In its ^1^H-NMR spectrum, two sets of closely spaced signals are observed indicating the existence of an isomeric mixture. Moreover, the NCH_2_ protons become diastereotopic in the carbene complex *cis*-**17** giving rise to a doublet for each proton (AA′ spin system) (Figure 11), whereas their resonances are detected as singlets in the precursor ^B^**T**·HBr. This observation is in agreement with a *cis*-arrangement of the two carbene ligands, in which a free rotation around the Pd-C bond is restricted due to the steric repulsion of the bulky N-thienylmethyl substituents. As a consequence, the two isomers have been assigned to *cis*-*syn* and *cis*-*anti* rotamers, in which the unsymmetrical carbene ligands differ in the orientation of their N-substituent. Notably, the presence of both rotamers is rarely observed in cis-configured complexes. Due to sterical reasons, the major set of signals is assigned to the *cis*-*anti* isomer. Correspondingly, two sets of signals are also observed in the ^13^C NMR spectrum with two carbene resonances at 174.5 and 174.2 ppm, which are in the typical range observed for *cis*-bis(benzimidazolin-2-ylidene) complexes of Pd(II) [8]. The molecular structure of *cis*-**17** obtained from X-ray diffraction analysis on single crystals revealed the sterically more favored *cis*-*anti* geometry. 

## 6. Thiolato-NHC Complexes

In comparison to the thioether group, the thiolato function is more electron rich and forms stronger metal sulfur bonds, which results in a versatile and diverse coordination chemistry. Although some methods of synthesizing thiolato-NHC complexes exist, they suffer from the limitation that air sensitive precursors, such as free thiols and metal(0) compounds, are required [19,20,21]. Furthermore, all reported examples are based on a saturated five- or six-membered N-heterocyclic ring system, while those based on benzimidazole and imidazole remain to be explored. Thus we explored simpler and more straightforward synthetic routes to thiolato-NHC complexes [11,12].

### 6.1. Thiolato-Bridged Pd(II) Complexes

**Scheme 11:**
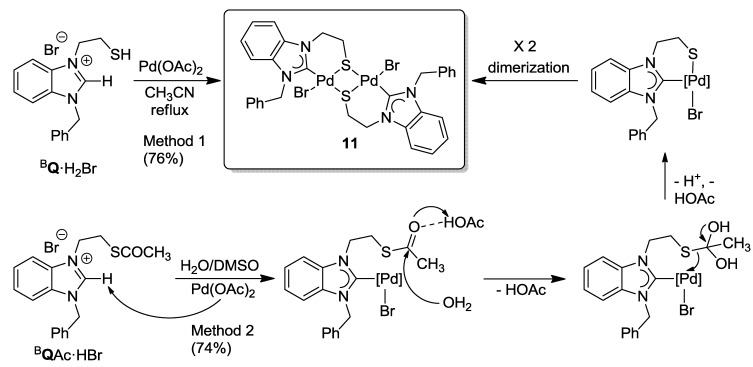
Synthesis of thiolato-bridged Pd(II) benzimidazolin-2-ylidene complex **11** [11].

**Figure 12:**
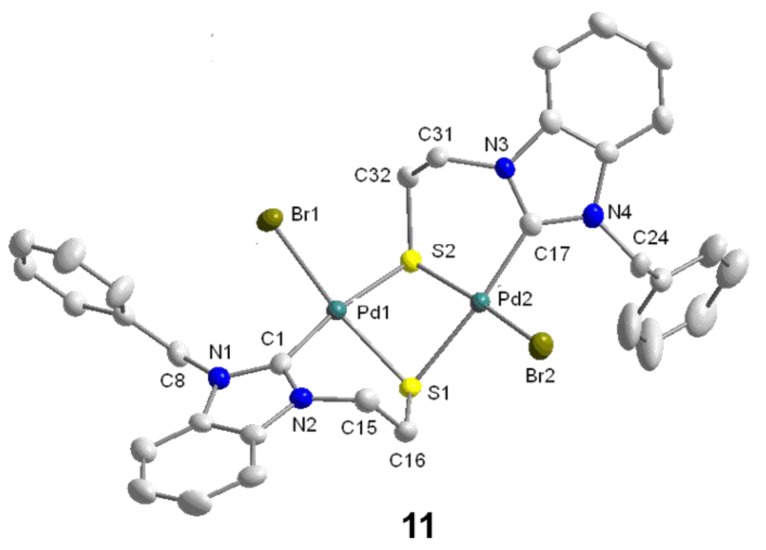
Molecular structure of **11** [11].

The reaction of thiol-functionalized salt ^B^**Q**·H_2_Br with one equiv of Pd(OAc)_2_ in degassed CH_3_CN under reflux yielded the thiolato-bridged dimeric Pd(II) benzimidazolin-2-ylidene complex [PdBr(^B^**Q**-*k*^2^*C*,*µ*-*S*)]_2_ (**11**) in a good yield of 76% (Scheme 11, method 1). More straightforwardly, it was found that the direct reaction of thioester salt ^B^**Q**Ac·HBr with one equiv of Pd(OAc)_2_ in DMSO at 80 °C also yielded complex **11** in a similar yield of 74% (Scheme 11, method 2). Apparently, the methyl-thioester-function undergoes *in situ* hydrolysis probably assisted by free HOAc liberated from the deprotonation of ^B^**Q**Ac·HBr with Pd(OAc)_2_. Alternatively, the hydrolysis may be activated by the coordination of the thioester to the Lewis acidic Pd(II) center. The resulting thiolate coordinates to the Pd(II) center, and subsequent dimerization affords the desired thiolato-bridged Pd(II) carbene complex **11**, as evidenced by NMR spectroscopy and its solid state structure determined by X-ray diffraction (Figure 12). In this reaction, the thioester-function acts as a thiol-protecting group simplifying the synthesis of thiolato-complexes to a great extent. Method 2 is preferred over Method 1, since it shortens the reaction sequence and also eliminates the requirement to work under an inert atmosphere.

**Scheme 12:**
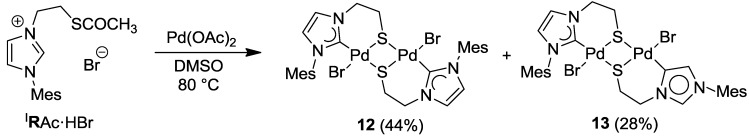
Syntheses of thiolato-bridged Pd(II) imidazolin-2-ylidene complexes [12].

Similarly, the imidazolium thioester salt ^I^**R**Ac·HBr was treated with one equiv of Pd(OAc)_2_ in DMSO at 80 °C, which afforded a mixture of thiolato-bridged dinuclear [Pd_2_S_2_] carbene complexes in an overall yield >72% (Scheme 12) [12]. Both the expected dimeric complex [PdBr(^I^**R**-*k*^2^*C*,*µ*-*S*)]_2_ (**12**) and its interesting constitutional isomer [Pd_2_Br_2_(^I^**R**-*k*^2^*C*,*µ*-*S*)(^I^**R**’-*k*^2^*C*,*µ*-*S*)] (**13**) were isolated. Compared to **12**, the two NHCs in **13 **adopt different coordination modes: one is bound to the Pd center *via* C(2) (normal bonding mode), while the other one coordinates *via* C(4) (*mesoionic *[22] bonding mode). The formation of the mesoionic carbene by competing deprotonation at C(4) probably helps to reduce the steric congestion caused by the bulky mesityl substituent. 

**Figure 13:**
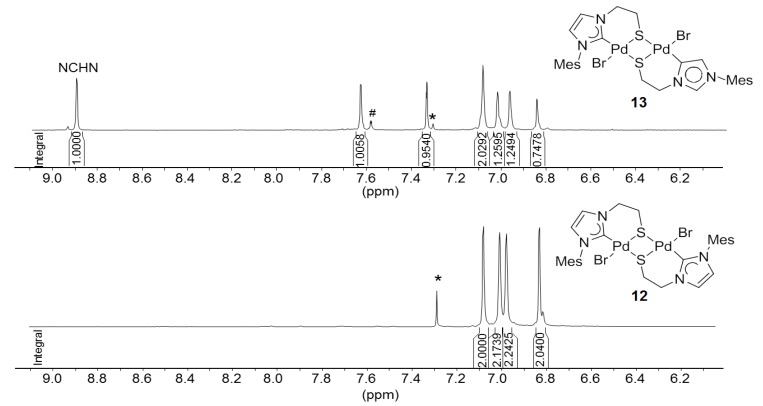
Comparison of aromatic signals of ^1^H-NMR spectra of **12 **(500 MHz, CDCl_3_) and **13** (500 MHz, DMSO-*d6*). The peaks marked with * correspond to solvents and the one with # is due to the impurity.

**Figure 14:**
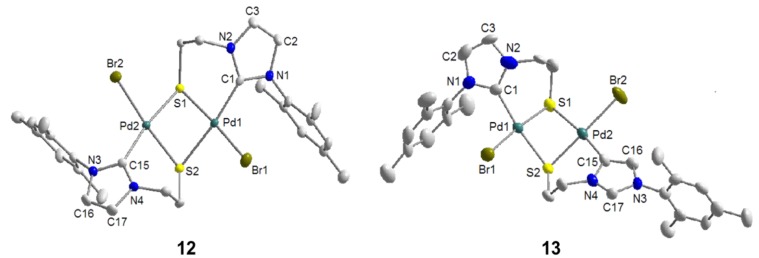
Molecular structures of **12** and **13 [12]**.

The formation of **13** is evidenced by NMR spectroscopy, which reveals distinct differences to complex **12** (Figure 13). Due to the unsymmetrical nature of compound **13** as a result of two different carbene ligands, its ^1^H- and ^13^C-NMR spectra are more complicated and exhibit twice the number of signals compared to those of its isomer **12**. More importantly, a sharp signal for the NCHN proton of the mesoionic carbene is still observed downfield at 8.90 ppm in the ^1^H-NMR spectrum. Furthermore, the formations of both **12** and **13** were evidenced by their solid state structures (Figure 14) determined by single crystal X-ray diffraction.

To the best of our knowledge, complex **13** is the first example of a dinuclear complex, in which a normal NHC and its mesoionic isomer are coordinating two different metal centers within the same complex. In principle, this allows for a direct comparison of the two isomeric binding modes and their impact on bond parameters around each metal center. For example, the Pd-S bond [2.378(3) Å] *trans* to the C(4) bound carbene is found to be longer than the one *trans* to the C(2) carbene [2.351(3) Å], implying a stronger *trans* influence of the former. 

### 6.2. Reactivity Studies with Ag-carboxylates

In order to investigate the robustness of the [Pd_2_S_2_] core of **11** and **12**, attempts were made to alkylate the thiolato-bridges [11], to expose the compound to Ag-salts [11] or to treat the complex with external thiolato ligands [12].

**Figure 15:**
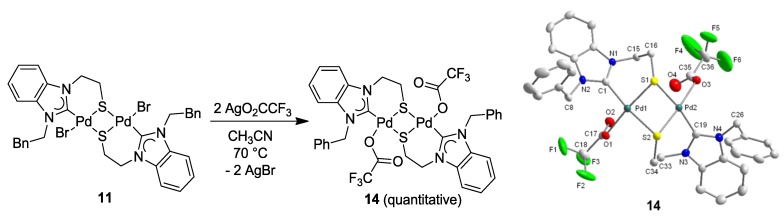
Synthesis of Pd(II) trifluoroacetato complex **14** and its molecular structure [11].

The metathesis reaction of mixed halo/NHC Pd(II) complexes with Ag-carboxylates is an established method to synthesize mixed carboxylato-carbene complexes as potentially useful catalyst precursors [23,24]. Complex **11** was reacted with 2 equiv of AgO_2_CCF_3_ in CH_3_CN (Figure 15), which cleanly afforded the dinuclear, mixed carbene-carboxylato Pd(II) complex [Pd(O_2_CCF_3_)(^B^**Q**-*k*^2^*C*, *µ*-*S*)]_2_ (**14**) in quantitative yield without affecting the [Pd_2_S_2_] core [11]. Although the potential interaction of the soft sulfur donor with the soft Ag(I) ion could compete with the desired halide abstraction, the bridging mode of the thiolato-donor efficiently reduces the number of lone-pairs available at sulfur, which prevents the interference with silver. The solid state structure of **14** confirms that both bromido ligands in **11** have been replaced by trifluoroacetato ligands with the [Pd_2_S_2_] core remaining intact (Figure 15).

### 6.3. Reactivity Studies with Alkylating Reagents

**Scheme 13:**
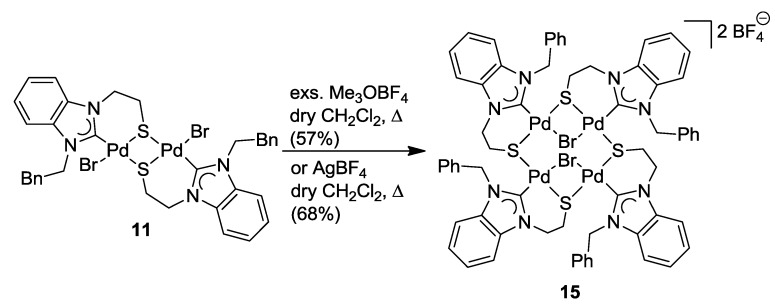
Synthesis of tetranuclear complex **15** [11].

The successful S-alkylation of thiolato-bridged complexes of type **11** would allow for a convenient template-directed synthesis of thioether-functionalized NHC complexes. Thus, a series of electrophiles, including iodomethane and dimethylsulfate, has been used in an attempt to alkylate the sulfur atoms of dimer **11**, however to no avail, and only starting materials were re-isolated. Again the lower electron density at the sulfur atoms as a result of the bridging mode may be the reason that prevents their alkylation. Unexpectedly, the reaction of **11** with an excess of the strong alkylating agent Me_3_OBF_4_ led to the formation of the novel tetranuclear complex [Pd_4_Br_2_(^B^**Q**-*k*^2^*C*,*µ*-*S*)_4_](2BF_4_) (**15**) (Scheme 13) [11]. Apparently, electrophilic attack occurred preferably at the bromido ligand, supposedly liberating CH_3_Br and resulting in a rearrangement of the unsaturated complex fragments to tetranuclear **15**.

To verify the unusual role of Me_3_OBF_4_ as a halide-abstracting agent, **11** was treated with one equiv of AgBF_4_. Indeed, this reaction furnished complex **15** as well. Consequently, Me_3_OBF_4_ can be regarded as a very useful metal-free alternative to the commonly used Ag- and toxic Tl-based reagents.

**Figure 16:**
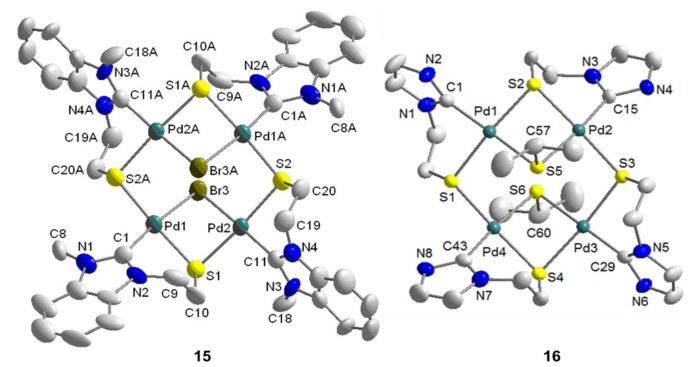
Molecular structures of tetranuclear complexes **15** and **16** [12].

The molecular structure of **15** shows an eight-membered square consisting of alternating four palladium and four sulfur atoms with dimensions of 4.653(5) Å · 4.662(5) Å (Figure 16). Each of the four Pd(II) centers is coordinated by one carbene, one *µ*-bromido, and two *µ*-thiolato ligands in a square-planar fashion. Two carbene moieties are each found above and below the [Pd_4_S_4_] macrocycle, and consequently, the two bridging bromido ligands *trans* to the carbenes are found at opposite sides.

### 6.4. Reactivity Studies with External Thiolato Ligands

**Scheme 14:**
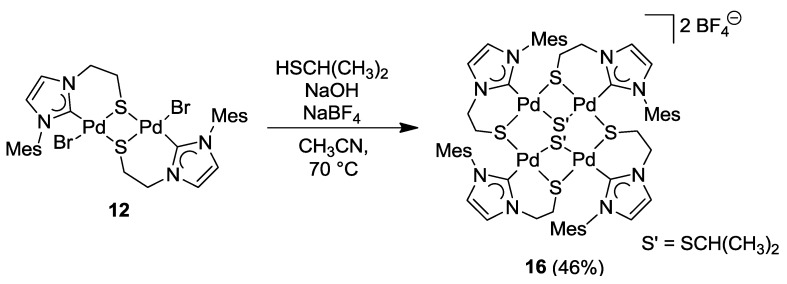
Synthesis of tetranuclear complex **16** [12].

The imidazolin-2-ylidene complex **12** was treated with two equiv of *in situ* generated sodium isopropyl thiolate. Initially, a simple bromido-thiolato ligand exchange was expected. Although the bromido ligands were indeed abstracted, a tetra-palladium species formed instead [12]. Reaction optimization *via* counter anion exchange using NaBF_4_ resulted in the isolation of the tetranuclear [Pd_4_S_4_] macrocyclic compound [Pd_4_{SCH(CH_3_)_2_}_2_(^I^**R**-*k*^2^*C*,*µ*-*S*)_4_](2BF_4_) (**16**) (Scheme 14). Seemingly, the bromido ligand in **12** was first replaced with one isopropyl thiolato ligand accompanied by the precipitation of NaBr. The new highly nucleophilic thiolato ligand replaces a 2nd bromido ligand of another Pd(II) center leading to a rearrangement of one Pd-S bond and subsequent dimerization to yield tetranuclear **16**.

As shown by the molecular structure of **16**, each Pd(II) center is surrounded by one carbene and three *µ*-thiolato donors in a square-planar fashion (Figure 16). Similar to the tetranuclear complex **15**, there is an eight-membered [Pd_4_S_4_] macrocycle with almost the same dimensions of [4.6421(24) Å · 4.6617(24) Å]. The two bridging isopropyl thiolato groups are located at different sides of the macrocycle.

## 7. Catalytic Applications

Despite the fact that sulfur functions are generally regarded as catalyst poisons [25], transition metal complexes with sulfur donors have found applications in catalyzing various reactions, such as allylic alkylations [26,27,28], hydroformylations [29], hydrogenations [30,31,32] and C-C coupling reaction [33]. In order to evaluate the catalytic activities of Pd(II) complexes bearing sulfur-functionalized NHCs, selected compounds synthesized in our study have been tested in Suzuki-Miyaura, Mizoroki-Heck, and hydroamination reactions.

**Table 1:** Suzuki-Miyaura coupling reactions *^a^* catalyzed by complexes **11**, **14**, and **15** [11]. 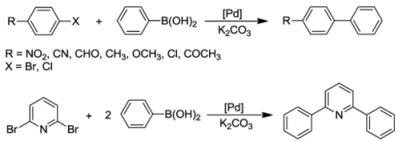

Entry	Catalyst	temp. [°C]	Aryl halide	catalyst loading [mol%]	t [h]	yield [%] *^b^*	TON	TOF [h^−1^]
1 *^c^*	**11**	60	4-bromobenzophenone	0.001	24	0	0	0
2	**11**	60	4-bromobenzophenone	0.001	24	84	84,000	3500
3	**14**	60	4-bromobenzophenone	0.001	24	>99	100,000	4167
4	**15 ** *^d^*	60	4-bromobenzophenone	0.001	24	48	96,000	4000
5	**14**	60	4-bromobenzonitrile	0.001	24	>99	100,000	4167
6	**14**	60	1-bromo-4-nitrobenzene	0.001	42	90	90,000	2143
7	**14**	80	4-bromobenzaldehyde	0.001	23	>99	100,000	4167
8	**14**	100	2,6-dibromopyridine *^e^*	0.0025	48	>99	20,000	417
9	**14**	100	4-bromotoluene	0.0025	20	97	38,800	1940
10	**14**	100	4-bromoanisole	0.0025	24	86	34,400	1433
11	**14**	100	1-bromo-4-chlorobenzene	0.0025	24	72 *^f^*	28,800	1200
12 *^g^*	**14**	100	4-chlorobenzaldehyde	0.1	72	41	410	5.7

*^a^* Reaction conditions: 1 mmol of aryl halide; 1.4 mmol of phenylboronic acid; 1.4 mmol of K_2_CO_3_; 1 mL of H_2_O; the desired amount of precatalyst. *^b^* Yields were determined by ^1^H NMR spectroscopy for an average of two runs. *^c^* The solvent is 1 mL of dioxane. *^d^* 0.0005 mol% of **15**. *^e^* 0.5 mmol of 2,6-dibromopyridine. *^f^* isolated yield. *^g^* With addition of 1.0 equiv of [N(*n*-C_4_H_9_)_4_]Br.

### 7.1. Suzuki-Miyaura Coupling Reactions

The thiolato-bridged dimeric complex **11** was first tested in Suzuki-Miyaura coupling of 4-bromo-acetophenone and phenylboronic acid at low catalyst loading (0.001 mol%) in H_2_O or dioxane. H_2_O proved to be a superior solvent as higher yields were obtained even under mild conditions (Table 1, entry 1 and 2), which is in line with previous findings [34,35,36,37]. A comparative study on the thiolato-NHC complexes **11**, **14** and **15** revealed that **14** bearing labile trifluoroacetato ligands was the best precatalyst (entries 2–4).

**Figure 17:**
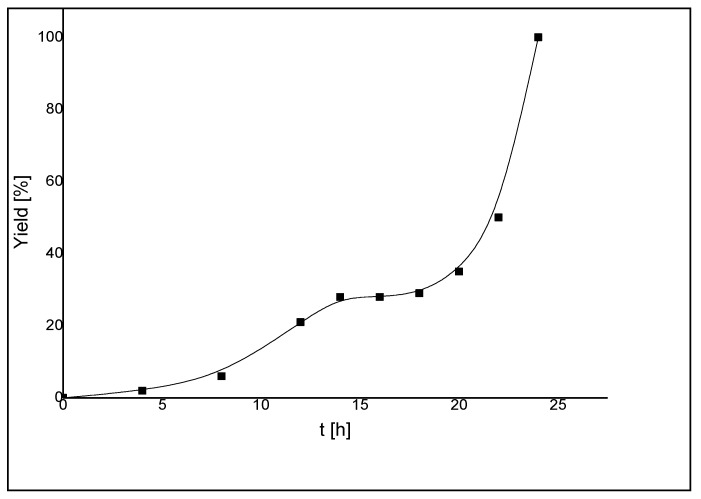
Concentration/time diagram [yield (%), time (h)] for the Suzuki-Miyaura coupling reaction of 4-bromoacetophenone and phenylboronic acid catalyzed by **14** [11].

In order to gain more insights into the catalytic behavior of **14**, a more detailed kinetic study was carried out on the coupling of 4-bromoacetophenone and phenylboronic acid. The concentration/time profile showed that the reaction experienced a long induction period of about 22 h before the rate increased exponentially leading to complete conversion within 2 h (Figure 17). A dimer-monomer equilibrium [38] before catalyst initiation was proposed to be responsible for the long induction time. Furthermore, the shape of the reaction profile suggested the involvement of various catalytically active species prior to the formation of the most active catalyst. 

The couplings of phenylboronic acid with various aryl halides catalyzed by **14 **were also studied. With aryl bromides, including 2,6-dibromopyridine, good yields were achieved (entries 5–11), whereas only a moderate yield was obtained with 4-chlorobenzaldehyde (entry 12). 

In order to compare the catalytic activities of both bis-*versus* mono-NHC complexes, selected S-Me functionalized complexes *cis*-**1a**, *trans*-**1a**, *cis*-**2a**, **3** and **4** were also tested in aqueous Suzuki-Miyaura couplings. These reactions gave good yields in the couplings of aryl bromides (Table 2), while only moderate to low results were obtained for chloro-substrates. Moreover, it was found that among bis(carbene) species, *cis*-**1a** and *cis*-**2a** performed slightly better than the *trans*-isomer *trans*-**1a**. The necessity for *trans*-**1a** to isomerize to its *cis* analogue during catalysis may account for the superior of *cis*-isomers [39]. However, the mixed NHC-phosphine complex **4** proved to be the most active. The thiophene-functionalized benzimidazolin-2-ylidene complex *cis*-**17** showed similar activities to those of the aforementioned bis(carbene) complexes in aqueous Suzuki-Miyaura reactions under identical conditions (Table 2, entries 6, 12, 18, 24, 30).

**Table 2:** Suzuki-Miyaura coupling reactions *^a^* catalyzed by **1a**, *cis*-**2a**, **3**, **4** and *cis*-**17** [8,13]. 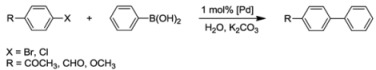

Entry *^a^*	Catalyst	Aryl halide	t [h]	temp. [°C]	yield [%] *^b^*
1	*cis*-**1a**	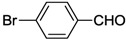	8	RT	>99
2	*trans*-**1a**				>99
3	*cis*-**2a**				>99
4	**3**				>99
5	**4**				>99
6	*cis*-**17**				>99
7	*cis*-**1a**	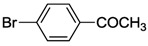	8	RT	98
8	*trans*-**1a**				84
9	*cis*-**2a**				90
10	**3**				85
11	**4**				>99
12	*cis*-**17**				85
13 *^c^*	*cis*-**1a**	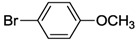	21	85	>99
14 *^c^*	*trans*-**1a**				>99
15 *^c^*	*cis*-**2a**				>99
16 *^c^*	**3**				>99
17 *^c^*	**4**				>99
18 *^c^*	*cis*-**17**				>99
19 *^c^*	*cis*-**1a**	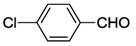	21	85	7
20 *^c^*	*trans*-**1a**				11
21 *^c^*	*cis*-**2a**				3
22 *^c^*	**3**				8
23 *^c^*	**4**				52
24 *^c^*	*cis*-17				9
25 *^c^*	*cis*-**1a**	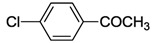	21	85	3
26 *^c^*	*trans*-**1a**				9
27 *^c^*	*cis*-**2a**				23
28 *^c^*	**3**				25
29 *^c^*	**4**				44
30 *^c^*	*cis*-**17**				14

*^a ^*Reaction conditions: 1 mmol of aryl halide; 1.5 mmol of phenylboronic acid; 3 mL of water; 2 equivalents of K_2_CO_3_; 1 mol% of precatalyst**. ***^b ^*Yields were determined by ^1^H-NMR spectroscopy for an average of two runs. *^c ^*With addition of 1.5 equivalents of [N(*n*-C_4_H_9_)_4_]Br.

### 7.2. Mizoroki-Heck Coupling Reactions

The benzimidazolin-2-ylidene CSC-pincer type Pd(II) complexes were tested in Mizoroki-Heck reactions. Pseudo-pincer complexes **5** and **6** as well as pincer complex **7** were found to be highly active in catalyzing the couplings of a range of activated substrates (Table 3, entries 1–15). 

**Table 3:** Mizoroki-Heck coupling reactions *^a^* catalyzed by complexes **5**–**7** [9]. 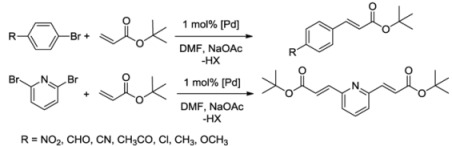

Entry	Catalyst	Aryl halide	t [h]	temp [°C]	yield [%] *^b^*
1	**5**	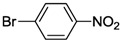	24	120	>99
2	**6**				>99
3	**7**				>99
4	**5**	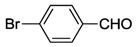	24	120	>99
5	**6**				>99
6	**7**				>99
7	**5**	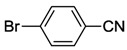	24	120	>99
8	**6**				>99
9	**7**				>99
10	**5**	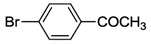	24	120	>99
11	**6**				>99
12	**7**				>99
13	**5**	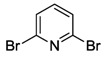	24	120	>99
14	**6**				>99
15	**7**				>99
16	**5**	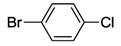	24	120	50
17	**6**				58
18	**7**				45
19 *^c^*	**5**	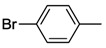	24	140	53
20 *^c^*	**6**				52
21 *^c^*	**7**				47
22 *^c^*	**5**	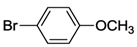	24	140	41
23 *^c^*	**6**				35
24 *^c^*	**7**				51

*^a^* Reaction conditions: 1 mmol of aryl halide (0.5 mmol of 2,6-dibromopyridine); 1.4 mmol of *tert*-butyl acrylate; 3 mL of DMF; 1.5 mmol of NaOAc; 1 mol% of precatalyst. *^b^* Yields were determined by ^1^H NMR spectroscopy for an average of two runs. *^c^* With addition of 1.5 equiv of [N(*n*-C_4_H_9_)_4_]Br.

Complex **7** could still give quantitative yield with a loading of as low as 10^-3^ mol% in the coupling of 4-bromobenzaldehyde. However, only poor to moderate yields were obtained with deactivated aryl bromides (Table 3, entries 16–24). Notably, a comparison did not reveal the superiority of any complex. It was anticipated that all three complexes decomposed to palladium nanoparticles of very similar size that do the catalytic work.

### 7.3. Hydroamination of Alkynes

The imidazole-based CSC-pincer type Pd(II) complexes, on the other hand, were studied in hydroamination reactions. The direct addition of a primary or secondary amine to a double or triple bond is a reaction of 100% atom efficiency, which is highly desirable and consistent with the current development of “greener” methodologies [40,41]. Since the investigations on Pd(II) NHC complexes catalyzed hydroaminations are rare [42,43,44], complexes **8**–**10** were studied in the intermolecular hydroamination reaction of phenylacetylene with various anilines. 

**Table 4:** Hydroamination reactions *^a^* catalyzed by complexes **8**–**10** [10]. 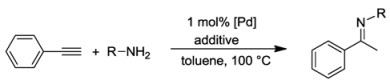

Entry	Aniline	complex 8 yield *^b^*	complex 9 yield *^b^*	complex 10 yield *^b^*
1	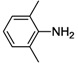	-	87 *^c^*	-
2	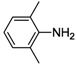	55	94	53
3	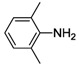	-	0 *^d^*	-
4	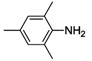	43	75	53
5	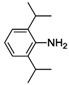	39	58	42
6	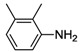	26	48	35
7	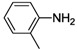	22	51	28
8	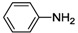	23	30	20

*^a^* *Reaction conditions*: 1.0 mmol of phenylacetylene; 0.5 mmol of aniline; 1 mL of toluene; 5 mol% of triflic acid; 1 mol% of precatalyst. *^b^* Yields were determined by GC-MS by using *n*-hexadecane as internal standard for an average of two runs. *^c^* With addition of 10 mol% of triflic acid. *^d^* Without addition of triflic acid.

A benchmark reaction with 2,6-dimethylaniline and 1 mol% of **9** showed that 5 mol% triflic acid was essential to get a good result (Table 4, entries 1–3). An extensive study on different anilines and catalysts revealed that good to moderate yields were obtained with mesitylaniline and phenylacetylene, and complex **9** outperformed **8** and **10** (entries 2 and 4). However, reactions with 2,6-diisopropyl-aniline, 2,3-dimethylaniline, 2-methylaniline, and the parent aniline gave only poor yields, indicating that anilines with intermediate steric bulk are best for this reaction (entries 5–8).

## 8. Conclusions and Outlook

A series of Pd(II) complexes bearing NHCs with different sulfur-functions, *i.e.*, thioether, sulfoxide, thiolato, and thiophene, have been synthesized. Experimental evidence for a true hemilabile coordination behavior was obtained using a thioether-NHC complex. Complexes with more rigid CSC-pincer type ligands have also been synthesized, and the reasons governing the pincer *versus* pseudo-pincer formation have been investigated. Incorporation of the thiolato function into Pd(II) complexes resulted in a diverse coordination chemistry giving rise to both dinuclear and tetranuclear complexes. The catalytic activities of selected complexes have been studied in Mizoroki-Heck, Suziki-Miyaura, and hydroamination reactions. Future studies may focus on complexation of these sulfur-functionalized ligands with other metal centers, e.g., Ni(II), Pt(II), and Ir(I), which should lead to complexes with enhanced structural diversities and interesting properties with potential applications in metallo-based supramolecular chemistry and catalysis.
